# Llama Antibody Fragments Recognizing Various Epitopes of the CD4bs Neutralize a Broad Range of HIV-1 Subtypes A, B and C

**DOI:** 10.1371/journal.pone.0033298

**Published:** 2012-03-15

**Authors:** Nika Strokappe, Agnieszka Szynol, Marlèn Aasa-Chapman, Andrea Gorlani, Anna Forsman Quigley, David Lutje Hulsik, Lei Chen, Robin Weiss, Hans de Haard, Theo Verrips

**Affiliations:** 1 Biomolecular Imaging, Department Biology, Faculty of Science, Utrecht University, Utrecht, The Netherlands; 2 Division of Infection and Immunity, UCL/MRC Centre for Medical Molecular Virology, University College London, London, United Kingdom; 3 Unit of Virus Host Cell Interactions (UVHCI), UMI 3265, Université Joseph Fourier-EMBL-CNRS, Grenoble, France; 4 Vaccine Research Centre, National Institute of Allergy and Infectious Diseases (NIAID), Nation Institutes of Health (NIH), Bethesda, Maryland, United States of America; The University of Hong Kong, Hong Kong

## Abstract

Many of the neutralising antibodies, isolated to date, display limited activities against the globally most prevalent HIV-1 subtypes A and C. Therefore, those subtypes are considered to be an important target for antibody-based therapy. Variable domains of llama heavy chain antibodies (VHH) have some superior properties compared with classical antibodies. Therefore we describe the application of trimeric forms of envelope proteins (Env), derived from HIV-1 of subtype A and B/C, for a prolonged immunization of two llamas. A panel of VHH, which interfere with CD4 binding to HIV-1 Env were selected with use of panning. The results of binding and competition assays to various Env, including a variant with a stabilized CD4-binding state (gp120_Ds2_), cross-competition experiments, maturation analysis and neutralisation assays, enabled us to classify the selected VHH into three groups. The VHH of group I were efficient mainly against viruses of subtype A, C and B′/C. The VHH of group II resemble the broadly neutralising antibody (bnmAb) b12, neutralizing mainly subtype B and C viruses, however some had a broader neutralisation profile. A representative of the third group, 2E7, had an even higher neutralization breadth, neutralizing 21 out of the 26 tested strains belonging to the A, A/G, B, B/C and C subtypes. To evaluate the contribution of certain amino acids to the potency of the VHH a small set of the mutants were constructed. Surprisingly this yielded one mutant with slightly improved neutralisation potency against 92UG37.A9 (subtype A) and 96ZM651.02 (subtype C). These findings and the well-known stability of VHH indicate the potential application of these VHH as anti-HIV-1 microbicides.

## Introduction

Neutralising antibodies against the human immunodeficiency virus type 1 (HIV-1) are powerful tools not only for understanding the virus structure [1]–[4] and the mechanism of cellular entry [5], [6], but also for passive immunization [7]–[9]. Many monoclonal antibodies specific for HIV-1 envelope proteins, gp120 and gp41, have been isolated both from immunised animals and infected individuals. However, only a few of these are broadly neutralising. These rare antibodies, including b12, 2G12, 2F5, 4E10 and ×5 [10], [11] have all been derived from HIV-1 subtype B infected patients and, besides 4E10, display limited activity against the globally most prevalent subtype C HIV-1 [12]–[15]. More recently other promising broadly neutralizing monoclonal antibodies (bnmAbs), notably PG16, PG9 [16], [17], HJ16 [18], VRC01-03 [19] and 3BNC60 and 117 [20] have been described. Many of these bnmAbs recognize the CD4bs and the sometimes relatively small differences in the interaction area, derived from X-ray data, resulted in quite different neutralization potencies [19], [21]. Isolation and characterisation of novel bnmAbs, with specific attention to non-subtype B viruses, may aid the design and development of a vaccine capable of inducing a broadly protective antibody immune response. Additionally, such antibodies might be developed as specific entry inhibitors for inclusion in HIV-1 microbicides [22].

Llamas, and other *Camelidae*, possess conventional antibodies and heavy chain antibodies. The latter are devoid of light chains [23] and the Variable domain of the Heavy chain of the Heavy chain antibodies (VHH) is therefore solely responsible for antigen recognition. The specificities and affinities of VHH are comparable to those of IgGs even though the size of a VHH is only approximately 15 kDa., compared to the 150 kDa. of IgG. On average, VHH have longer complementarity determining regions 3 (CDR3) [24]–[26], a feature that might facilitate binding into deeper cavities on the antigen surface. Grooves and cavities play a crucial role in multiple biological activities as these often form the specific interaction site between two molecules [25]. Fitting into the CD4bs is thought to be important for potent neutralisation of HIV-1 via binding to the envelope spike [16], [27]. Moreover the small size of VHH may be an important property to inhibit transmission of HIV in the small viral synapsis [28]. The high stability [24], [29]–[32] and the often excellent expression yield of VHH in microbial fermentations [33], [34] make VHH realistic candidates for the development of microbicides to protect against HIV infections.

We have shown that neutralising VHH can be raised in llamas immunized with gp120 of HIV-1_CN54_ [35]. Although the selected VHH exhibited neutralising effects against HIV-1 primary isolates of subtype B and to a lesser extent subtypes C, they did not neutralise HIV-1 subtypes A, A/G and D.

In the present study, we immunised two llamas with a mixture of two different antigens, gp140_CN54_ (subtype B/C) and gp140_UG37_ (subtype A) to promote the development of broadly reactive VHH. Here we describe the selection of VHH from immune phage display libraries by competition with sCD4, which resulted in the isolation of a number of VHH that not only competed with broadly neutralising anti-CD4 binding site antibody (b12) for binding to HIV-1 envelope proteins, but also revealed neutralising activities against a panel of primary HIV-1 including A, B and C subtypes. We classified the neutralising VHH into three groups based on sequence analysis and alignment against the llama's germline V-, D- and J-genes, binding and competition experiments, and neutralization assays. These data demonstrate the diversity of epitopes recognized by these VHH and suggest various mechanisms of HIV-1 entrance inhibitions.

## Materials and Methods

### Ethics statement

The prolonged Llama immunizations were approved and performed according to the guidelines of Utrecht University Animal Ethical Committee (approval ID: 2007.III.01.013).

### Proteins

Soluble CD4 (sCD4) was purchased from R&D systems (cat 514-CD/CF), Concanavalin A (ConA; cat. C2010) and biotinylated ConA (cat. C2272) were purchased from Sigma-Aldrich Chemie BV, The Netherlands. Recombinant HIV-1 envelope proteins gp140_CN54_ (0699), gp120_IIIB_ (0607) and gp120_CN54_ (6015) used for binding and competition assays, and the anti-CD4bs monoclonal antibody (mAb) b12 (3065) were obtained from the Centre for AIDS Reagents, NIBSC HPA UK, supported by the EC FP6/7 Europrise Network of Excellence, and NGIN consortia and the Bill and Melinda Gates GHRC-CAVD Project and were donated by Polymun, Immunodiagnostics, Immune Terchnology and Drs D P Burton and P Parren, respectively. Envelope proteins gp140_UG37_ and gp140_CN54_ used for immunization were kindly provided by Dr S. Jeffs, Wright-Fleming Institute, Division of Medicine, Imperial College London, London, UK. Envelope proteins gp120_YU2_ and its modified variant gp120_Ds2_ were kindly provided by Dr. P. Kwong, Vaccine Research Center, National Institute of Allergy and Infectious Diseases, National Institutes of Health, Bethesda, USA. The anti llama IgG_3_ mAb 8E1 was kindly provided by BAC BV, Naarden.

### Viruses

HIV-1 IIIB (0101) and MN (0102) were obtained from the Centre for AIDS Reagents, NIBSC HPA UK, supported by the EC FP6/7 Europrise Network of Excellence, and NGIN consortia and the Bill and Melinda Gates GHRC-CAVD Project and was donated by *Drs R Gallo & M Popovic* and propagated in H9 and C8166 cells, respectively. Virus stocks of HIV-1 envelopes pseudotyped with the pSG3Δenv vector and replication-competent HIV-1 molecular clones were produced by transfection of 293T cells [36]. The subtype B (THRO4156.18, TRJO4551.58, 6535.3) and C (Du156.12, Du422.1, ZM197M.PB7, ZM214M.PL15, ZM233M.PB6, ZM109F.PB4, ZM135M.PL10a, CAP45.2.00.G3) HIV-1 Reference Panels of Env. Clones [36] were obtained through the AIDS Research and Reference Reagent Program (ARRRP), Division of AIDS, NIAID, NIH (USA). HIV-1 subtype CRF02_AG (T257-71, T266-60, T278-50 and T33-7) gp160 clones, subtype CRF07_BC gp160 clones (CH038.12, CH064.20, CH091.9, CH110.2 and CH181.12), 96ZM651.02 and MS208.A1 p160 clones were kindly provided by Dr D. Montefiori (Duke University Medical Centre, Durham, USA) through the Comprehensive Antibody Vaccine Immune Monitoring Consortium (CA-VIMC) as part of the Collaboration for AIDS Vaccine Discovery (CAVD). Virus 92UG037.A9 is a gp120 clone of the primary isolate 92UG37 [37] cloned into the pHXB2Δenv vector [38].

### Binding sCD4 and b12 antibodies to gp140 and 120 molecules

To determine the functionality of envelope molecules, their interactions with sCD4 and b12 were tested. MaxiSorp microtitre plates (cat 442404, Nunc GmbH & Co. KG, Germany) were directly coated with envelope proteins serially diluted in PBS and incubated at 4°C overnight. After treatment with 4% skimmed milk powder (Marvel) in PBS (4% MPBS) for 1 h at room temperature (RT), 50 µL sCD4 [3 µg/mL] or 50 µL b12 [100 ng/mL] in 1% MBPS was added and incubated for an additional 1 h at RT, shaking. Soluble CD4 was detected with L120 (mouse anti CD4, 1∶10,000 in 1% MPBS; NIBSC) and b12 was detected with rabbit anti-human IgG (1∶10,000 in 1% MPBS; DAKO). Finally, peroxidase-conjugated donkey anti-mouse or donkey anti-rabbit IgG (1∶5,000 in 1% MPBS; Jackson Immunoresearch, West Grove, PA, USA) were added. Plates were washed three times with PBST (PBS supplemented with 0.05% Tween 20) between each step. The complexes were visualised by *o*-Phenylenediamine (OPD) according to method described by Verheesen *et al*. [39].

### Immunisation of *Lama glama* with gp140_UG37_ and gp140_CN54_

Two *Lama glama* were injected intramuscularly with mixture of gp140_CN54_, and gp140_UG37_, 50 µg of each protein in commercially available Stimune adjuvant (CEDI Diagnostics, Lelystad, The Netherlands). First boosting was given on day 7, with the same immunogens doses as the first injection. The following booster injections were given on days 14, 21, 28, 35 and 113 with mixture containing 25 µg of each gp140. Ten millilitres blood samples were collected at days 0 (before injection), 21 and 113. To construct immune libraries, 150 ml blood samples were collected at day 43 and 122.

To assess the llamas' immune response, MaxiSorp microtitre plates were coated with 50 µL gp140_CN54_, gp140_UG37_ or gp120_IIIB_ [5 µg/mL] as described above. After blocking with 200 µL 4% MPBS serial dilutions of pre-immune and immune sera were incubated for 1 h. Detection of bound llama single chain antibodies was performed by incubation with the IgG3 specific mAb 8E1 [40] followed and peroxidase-conjugated donkey anti-mouse all in 50 µL. Complexes were detected as described above.

### Phage library construction

To construct immune libraries, 150 ml blood samples were collected at 122 day, and peripheral blood lymphocytes (PBLs) were purified by Leucosep (cat 227290, Greiner Bio-One BV, The Netherlands). Total RNA was extracted from PBLs as described by Chomczynski *et al.* [41] and random primed complementary DNA (cDNA) was synthesised using SuperScript™ III First-Strand Synthesis System for RT-PCR (Invitrogen, cat. 18080-051). After purification of the cDNA with QIAquick PCR Purification Kit (Qiagen, cat 28106), the cDNA was used as template for PCR using the combination of the leader and CH2 based primers [39] which resulted in an amplification of the conventional and heavy-chain IgG repertoire gene fragments. Due to the lack of C_H_1 region in heavy-chain antibodies, the amplified gene fragments of conventional and heavy-chain antibodies were separated on agarose gel. Subsequently, a *Sfi*I restriction site was introduced upstream of FR1 in a nested PCR using the gel purified heavy chain amplicon as template. Since a *Bst*EII restriction site naturally occurs in 90% of the FR4 of llama heavy-chain antibodies genes [42] the repertoire of PCR-amplified genes was cut with *Bst*EII and *Sfi*I and the resulting 300–400 bp fragments were purified from agarose gel. Finally cDNA fragments were ligated into a phagemid vector for display on filamentous bacteriophage [43] and electroporated in *E. coli* TG1 (K12, _(*lac-pro*), *supE*, *thi*, *hsdD5/F′traD36*, *proA+B+*, lacIq, *lacZ*_M15).

The rescue with helper phage VCS-M13 and polyethylene glycol precipitation was performed as described previously [44]. Phage stock containing 5×10^11^ pfu/ml was prepared and used for subsequent biopanning.

### Selection of clones competing with sCD4 for binding to gp140

To select phages that specifically bound to CD4 binding site of gp140 the modified competitive elution method [35], [45], [46] using sCD4 as selective eluant was applied. Wells of MaxiSorp microtitre plates were coated with 100 µL gp140_CN54_ [2.5 or 0.5 µg/mL] in PBS overnight at 4°C. Blocking was performed with 2% MPBS. After washing the plate with PBS, 5×10^9^ phages, which were preincubated in blocking buffer for 30 min at RT, were added to the wells and incubated for 2 hours at RT. Next, the coated wells were extensively washed with PBS. Subsequently, 100 µL sCD4 [30 µg/mL] or 100 µL triethylamine (TEA) 100 mM were added and the plates incubated for 30 min at RT. The eluates were removed, the TEA eluted phage was neutralised with half volume of 1 M Tris pH 7.5, and subsequently 10-fold serial dilutions in PBS were prepared. Ten microlitres of each dilution was used for infection of 190 µL log-phase *E. coli* TG1. After infection at 37°C for 30 min without shaking, 5 µL of bacterial suspensions were spotted on LB agar plates supplemented with 100 µg/mL ampicillin and 2% glucose (LB/Amp^100^/Glu^2%^) to determine the enrichment of the first round. Moreover, 75 µµL of eluate was used for infection 0.5 mL log-phase *E. coli* TG1 to rescue phages [44], and were subsequently applied for second round of selection. The conditions of the following selection round were identical to the first one.

### Screening ELISA

At the end of the second round, 100 µL serially diluted infected *E. coli* TG1 were plated on LB/Amp^100^/Glu^2%^ agar plates and single colonies were picked and grown in 2× YT broth containing 100 µg/mL ampicillin and 2% glucose (2× YT/Amp^100^/Glu^2%^) in 96-well microtitre plate format.

Expression of the VHH from single clones was performed in 96 deep-well plates (cat.AB-0932, Westburg B.V, The Netherlands) according to the modified method described by Marks *et al*. [44]. Briefly, 1 mL of 2× YT/Amp^100^/Glu^0.1%^ broth was inoculated with 10 µL overnight culture and grown with shaking at 37°C until OD_600_ = 1 was reached. Expression of the protein was induced by adding IPTG (final concentration of 1 mM) and the cultures were grown for additional 4 hours with shaking at 37°C. After harvesting bacteria by centrifugation for 15 min at 4566×g and freezing pellets overnight at −20°C, bacteria were resuspended in 100 µL PBS and shaken for 2 h at 4°C. Next, spheroplasts were harvested by centrifugation for 15 min 4566×g at 4°C and supernatants (i.e. periplasmic fractions) containing VHH were taken for screening assays.

Periplasmic fractions were screened for their ability to interfere with binding of monoclonal antibodies (mAb) b12 to gp140_CN54_ by direct competitive enzyme-linked immunosorbent assay (ELISA). This approach was chosen because of the weak interaction between gp140_CN54_ and sCD4 in our ELISA setup that prevented screening of individual clones for competition with sCD4. For this purpose, wells of MaxiSorp microtitre plates were coated with 50 µL b12 [2 µg/mL] in PBS overnight at 4°C. Next, the b12-coated wells were blocked with 4% MPBS for 1 hour. In the meantime, mixtures of 5-fold diluted periplasmic fractions and 1 µg/mL gp140_CN54_ (final concentration) in 1% MPBS were prepared and incubated for 1 hour at room temperature. Then 50 µL of the mixtures were transferred into blocked, b12-coated wells and incubated for an additional 1 hour. To detect bound, non-inhibited gp140_CN54_ biotinylated concanavalin A (ConA) was used at concentration 2 µg/mL in 1% MPBS followed by addition of streptavidin-HRP conjugate. Complexes were detected as described above. Positive clones, which gave a low signal in the b12 competition assay, were selected and one-way sequencing was performed by application M13Rev primer [39] (ServiceXS, Leiden, The Netherlands). For further characterisation, VHH genes were recloned into the *E.coli* production vector and after expression the VHH were purified by means of immobilised metal affinity chromatography (IMAC) as it has been described by Verheesen *et al.* [47].

### Characterisation of selected VHH

#### Sequence comparisons

To analyse the maturation and to classify the selected VHH, we grouped them according to the use of the germline V, D and J segments using a database with twenty three different V gene segments of *Lama glama*; seven D gene segments of *Lama pacos;* and five J gene segments of *Lama glama* [48] supplemented with two missing J gene segments of *Lama pacos* [49]. To analyse the sequences, WHAT IF's [50] implementation of combined DNA codon/amino acid alignments of all germ line genes against selected VHH sequences was performed. The D gene segments were translated in three readings frames and the best fitting D-segment was used to analyse maturation of CDR3. The DNA/amino acid sequence format dictates that the triplet and the corresponding amino acid (so each codon is followed by its cognate amino acid e.g. cagQgtgVcagQ) remained associated in-phase in subsequent alignment procedures. The aligned DNA/amino acid sequences allowed fitting short sequences, such as D gene segments, and differentiate between silent and functional mutations.

#### VHH binding to different envelope proteins

To test binding of purified VHH to various envelope proteins, gp120_IIIB_ (clade B), gp140_CN54_ (its gp120 is representing clade C), gp140_UG37_ (clade A) gp120_YU2_ (clade B) and its modified variant gp120_Ds2_ were directly coated on Nunc MaxiSorp plates in 50 µL PBS, [5 µg/mL] by overnight incubation at 4°C. After blocking with 4% MPBS as described above, VHH diluted in 1% MPBS were allowed to interact with the envelope protein. Subsequently, bound VHH were detected with mouse anti-C-Myc (9E10) antibodies, which recognised C-terminal Myc tag incorporated in VHH, and the detection was followed by incubation with donkey anti-mouse – HRP conjugate. The signals were quantified by colorimetric assay described above. The experiments were performed in triplicates. Binding activities of 2E7 variants were tested in a similar manner, with exception of coating wells with 50 µL [4 µg/mL] of envelope proteins and detection of VHH with rabbit anti-llama VHH serum [51] followed by goat anti-rabbit HRP conjugate.

#### Competition assays

To test possibility of epitope overlapping of selected VHH and b12 monoclonal antibodies we applied the method described by Kuroki [36], [52]. Microtitre plates were coated with 50 µL, [2 µg/mL] of b12. Equal volumes of 2 µg/mL gp120_IIIB_, gp140_CN54_ or gp140_UG37_ and serially diluted VHH were preincubated, and subsequently added to the mAb b12 coated and blocked wells. After 1 hour, bound HIV-1 envelope proteins were detected by ConA as described above. The experiments were performed in triplicates. The absorbance at 490 nm (A_490_) of each tested sample was expressed as a percentage of positivity (PP), calculated by the following equation: PP = 100 * ((A_490sample_−A_490min_)/(A_490max_−A_490min_), where A_490min_ is a A_490_ of sample without VHH and without envelope protein; and A_490max_ is A_490_ of samples without VHH for particular envelope protein and finally A_490sample_ is the signal of sample with both the VHH and the envelope protein [53] [54]. To determine the descriptive measures such as mean (X) and standard deviation (S.D.), the results (expressed as PP) were processed with SPSS version 16.0 for Windows.

Cross-competition assays between selected VHH were performed in a similar manner. The only exceptions were: coating plates with 50 µL [3 µg/mL] of VHH and preincubation of equal volumes of 50 µg/mL VHH with 5 µg/mL gp140_CN54_ or gp140_UG37_. As a negative control we used anti EGFR VHH [42]. The experiments were performed in duplicates. PP was calculated as described above with the difference that A_490 min_ is a A_490_ of sample, where the same VHH were used in solution as the immobilized VHH; and A_490max_ is A_490_ of samples, where anti EGFR VHH were applied as the VHH in solution for the particular immobilized VHH.

### HIV neutralisation assay

The HIV-1 neutralising activities of the VHH were assessed in the TZM-bl cell based assay, as described previously [55] Briefly, 3-fold serial dilutions of purified VHH starting from 50 µg/mL were performed in duplicate in 10% (v/v) fetal calf serum (FCS) supplemented DMEM growth medium (Invitrogen, Paisley, UK). 200 TCID_50_ of virus was then added to each well and the plates were incubated for 1 hour at 37°C. TZM-bl cells were subsequently added (1×10^4^ cells/well) in growth medium supplemented with DEAE-dextran (Sigma-Aldrich, St Louis, MO, USA) at a final concentration of 15 µg/mL. Assay controls included replicate wells of TZM-bl cells alone (background control), and TZM-bl cells with virus assayed (virus control). No virus inactivation was observed with a negative control VHH. Following 48 hours incubation at 37°C, all 100 µL of the assay medium was removed and 100 µL of Bright-Glo luciferase reagent (Promega, Madison, WI, USA) was added to each well. The cells were allowed to lyse for at least 2 minutes, and the luminescence was then measured using a luminometer. The 50% inhibitory concentration (IC_50_) titres were calculated as the VHH concentration that achieved a 50% reduction in relative luminescence units (RLU) compared to the virus control RLU, after subtraction of the background control RLU from both values. The calculations were performed using the XLFit4 software (ID Business Solutions, Guildford, UK).

### Construction, expression and purification of VHH variants

Alanine scan was performed on residues N27, V29 (CDR1); D32 (FW2) and Y98, Y99, G100, R100b, Y100c, D101, Y102 (CDR3) of 2E7 by site-directed mutagenesis (SDM) with application of the QuikChange® Site-Directed Mutagenesis Kit (Stratagene, La Jolla, CA, USA) according to instructions provided by manufacturer. In addition, the same method was applied for introduction of back-to-germline-mutations in CDR1: N27S, V29F, and in FW2: D32N. The introduced mutations were verified by plasmid sequencing (ServiceXS, Leiden, The Netherlands). Expression and purification of mutated 2E7 variants were performed as previously described by Verheesen *et al*. [47].

## Results

### Immune response and library construction

**Figure 1:**
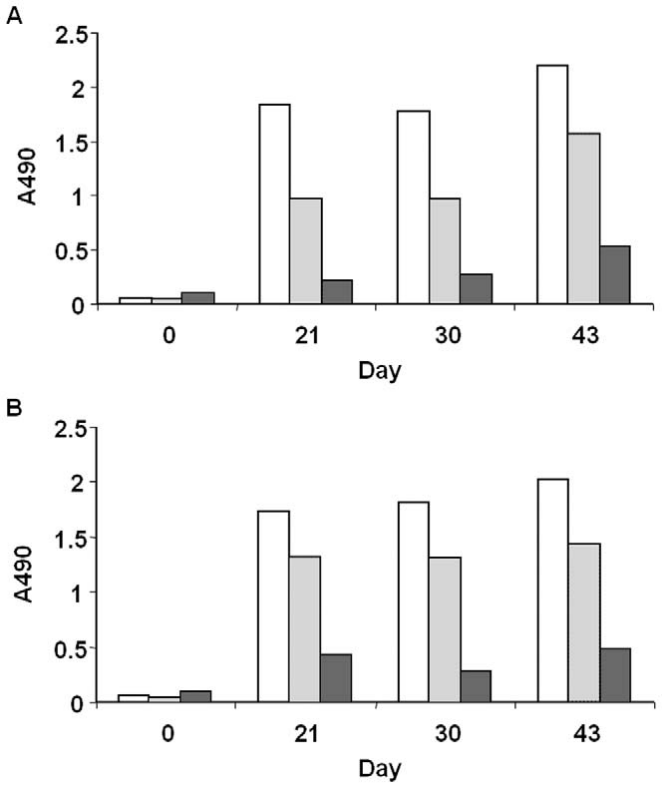
Heavy chain antibody response of llama 8 and 9. Heavy chain antibody response in llama 8 (A) and llama 9 (B) to gp140_UG37_ (□), gp140_CN54_ (▪), gp120_IIIB_ (▪) at indicated days following initial immunisation. Sera from llamas were collected, diluted 1000 fold and tested by ELISA for the presence of specific IgG_3_ heavy chain antibodies coated recombinant HIV-1 envelope proteins.

To obtain VHH specific for HIV-1 envelope proteins, two *Lama glama* were immunized with a cocktail of gp140_CN54_ (subtype, B/C, but the gp120 is representing subtype C) and gp140_UG37_ (subtype A). The induction of a humoral immune response was followed by testing sera of the animals before and after immunization by ELISA. Immunizations resulted in the induction of a specific heavy chain antibody response towards both immunogens (Fig. 1.). The titer of anti-gp140_UG37_ antibodies in immune sera was slightly higher than of anti-gp140_CN54_. In addition, the immune sera were also reactive with gp120_IIIB_ (subtype B). These data clearly demonstrate the successful induction of a humoral immune response towards the HIV-1 envelope proteins.

Since the immune response was good, library construction was continued. The synthesis of the VHH repertoires resulted in two libraries: llama 8 and llama 9, of approximately 10^7^ transformants each.

### VHH selection by competitive elution with sCD4

The specific competitive elution method required binding of sCD4 to gp140_CN54_. Therefore we tested how well the envelope protein was recognised by sCD4 in ELISA. As shown in supporting figure 1, sCD4 bound to gp140_UG37_ in a dose dependant manner (0.37–10 µg/mL). However, binding of sCD4 to gp140_CN54_ was only detectable at high concentrations (10 µg/mL) of the envelope protein. Therefore a high concentration of sCD4 was used as elution method during phage display, to ensure that all CD4 binding sites of the coated envelope proteins were saturated, thus preventing rebinding of phages displaying VHH that recognize this site.

Two rounds of selection were performed. In the first round, 1.5×10^6^ phages were non-specifically eluted by TEA from gp140_CN54_ coated at 2.5 µg/mL and 100 fold lower outputs were obtained from 0.5 µg/mL. Approximately 15 fold lower number of phages was eluted with sCD4 from 2.5 µg/mL coat and approximately the same number of phages were found for the 0.5 µg/mL coated wells as compared to TEA elution. For the second round, the rescued phages from 2.5 µg/mL gp140_CN54_ eluted with sCD4 were used and the selection procedure was repeated. Surprisingly no difference was observed between number of phages eluted with sCD4 and with irrelevant protein BSA for llama 8 library. However, in case of llama 9 library, ten times more phages were eluted from 2.5 µg/mL gp140_CN54_ with sCD4 than with BSA, thereby indicating a successful competitive elution with sCD4. Out of 280 single clones picked from the selection approximately 87% of the clones were able to bind specifically to gp140_CN54_.

In order to narrow down the investigation to clones that potentially were able to bind to CD4bs we performed a competition assay with b12. The setup of the sCD4 competition ELISA on gp140_CN54_, applied for selection of VHH with the specific competitive elution method, could not be used for screening purpose, because a too high concentration of sCD4 was required (10 µg/ml) to get a signal. Therefore we tested the ability of b12 to bind to the envelope proteins, since the b12 binding epitope overlaps partially with CD4bs [2]. In contrast to sCD4 binding, b12 binding to gp140_UG37_ was observed at envelope protein concentrations above 3.3 µg/mL (Fig. S1). For gp140_CN54_, a decent signal was already observed at concentrations as low as 1 µg/mL. Therefore, this setup was used as the competition ELISA for screening purpose. Approximately 11% from the 280 clones were able to compete with b12 (data not shown) and therefore selected for further VHH characterisation.

### Sequences analysis

**Figure 2:**
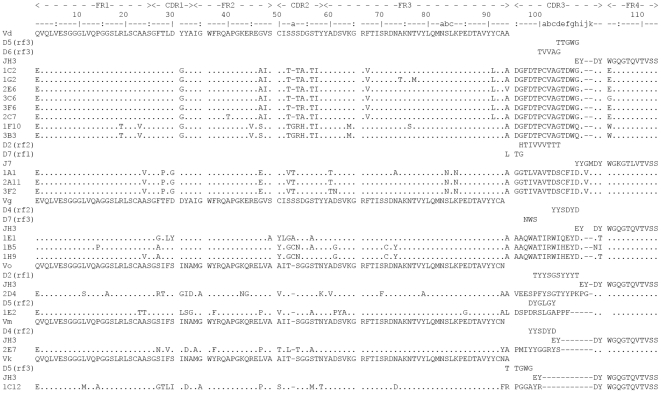
Alignment of the VHH against the germline V, D and J segments. Amino acids sequences of HIV-1 envelope protein CD4bs specific VHH aligned against *Lama glama* V and J germ line genes and D germ line genes of *Lama pacos*. The reading frame of the most likely D germline genes are marked. Numbering of amino acid according to Kabat *et al*.. CDRs of VHH are redefined.

The 30 competing clones were sequenced and based on deduced amino acids sequence 17 unique VHH were found, which were divided into seven families based on the DNA/amino acid alignment with the 23 V, the 7 D and the 7 J genes [48], [49] (Fig. 2). The maturation analysis revealed that four germline V gene segments were used to encode the VHH identified in the mAb b12 competition assay. Eleven VHH were derived from V_d_ gene, three VHH from V_g_, two from the V_o_ gene and another one from the V_m_ gene. Probably two different D genes have been used in the group derived from V_d_ gene and on that basis two subpopulations of VHH could be distinguished in this group. Sequences of all selected VHH revealed that only J3 or J7 gene segments contributed in the formation of the VHH CDR3 loops. A notable feature of the selected VHH is a relatively long CDR3 loop in most of the selected VHH. This panel of VHH has an average length of 16.4 residues compared to an average of 12.7 for the human heavy chain CDR3 and 8.5 for mice [56]. Examination of amino acids sequences showed that besides strictly conserved disulfide bridge (C22–C92) typical of the immunoglobulin fold, an extra pair of cysteines is present in the sequences of most of the newly selected VHH. The VHH originated from the V_d_ gene have an extra cysteine at position 50, which forms an S-S bridge with a cysteine at various positions in CDR3. This feature has been previously observed by Vu *et al.* [26]. Noteworthy is the fact that VHH 1B5 and 1H9, which derive from V_g_ gene segment, contain a cysteine at position 52a in CDR2 and a second additional cysteine at position 71 in FW3. Interestingly, both cysteines were not present in the original germline sequence, and were never seen before in our hands, nor in the antibody database Fungen (fungen.wur.nl) or the protein data bank (www.wwpdb.org). The introduction of this new location of a cysteine bridge must be important for the function of these VHH. Evidence for such maturation step is provided by 1E1, which is closely related to 1B5, but lacks the additional cysteines and is less potent than 1B5, indicating that the S-S bridge is important for binding and neutralization. This observation was confirmed by replacement of both cysteines in 1B5 (data not shown).

### Binding of selected VHH to envelope proteins

Purified VHH were tested in ELISA to characterise their ability to recognise directly coated HIV-1 envelope proteins gp140_CN54_, gp140_UG37_, gp120_IIIB_, gp120_YU2_ and its modified variant gp120_Ds2_ (Table 1, Fig. S2). All selected VHH bound reasonable well to both gp140_CN54_ and gp140_UG37_, with the exception of 2D4, which demonstrated limited binding to gp140_CN54_. VHH 1F10 and 1C2, as well as 1B5 and 1H9, which belong to two different families, were the best binders to gp140_CN54_ and gp140_UG37_ and reached half of maximal signal at concentrations below 0.63 µg/mL (or 42 nM). Only VHH of the 1B5 family (1B5, 1H9, 1E1) were able to bind to subtype B envelope proteins, although less efficiently than to gp140_CN54_ and gp140_UG37_. VHH 1B5 and 1H9 reached half of maximum signal at a concentration between 82 and 330 nM of all subtype B envelope proteins tested. In contrast, 1E1 lacking the second S-S bridge present in the other members of this family bound very weakly to gp120_IIIB_ as well as to gp120_YU2_, and binding to gp120_Ds2_ was not detectable. As a comparison, previously selected VHH A12 [35] bound to gp120_IIIB_ as good as 1B5 and 1H9, but was unable to bind to gp120_Ds2_ (data not shown). Furthermore, VHH A12 bound to gp140_UG37_ very well but poorly to gp140_CN54_.

**Table 1:** Summary of VHH binding to HIV-1 envelope proteins.

	HIV-1 envelope protein				
VHH	gp140_UG37_	gp140_CN54_	gp120_IIIB_	gp120_YU2_	gp120_Ds2_
1C2	+++	+++	-	-	-
1F10	+++	+++	-	-	+
1E1	++	+++	+	+	-
1B5	+++	+++	++	++	++
1H9	+++	+++	++	++	++
1E2	++	++	-	-	-
2E7	++	++	+	-	-
A12	+++	+	+++	N.D.	N.D.

The amount of VHH required to give half-maximal A_490_ was estimated from the respective binding curves. +++ half-maximal binding at <0.63 µg/mL; ++, 0.63–10 µg/mL; +, >10 µg/mL; -, no binding was observed even at the highest amount of VHH. N.D., not done.

### Competition assay between b12 and VHH

To assess whether the selected VHH bind to HIV-1 envelope proteins in a way that they may interfere with CD4 binding, we performed a competition assay with b12 (Table 2, Fig. S3). We chose to work with the anti-CD4bs mAb instead of sCD4, because of the poor sCD4 binding to gp140_CN54_ shown previously (Fig. S1). Note that the epitope of b12 does overlap with the CD4bs but it is not exactly the same, so competition with b12 does not necessarily mean competition with CD4. The competition with b12 was assessed by using gp120_IIIB_, gp140_CN54_ and gp140_UG37_, as it is known that CD4 binding sites may differ between various HIV-1 subtypes [2], [57]. Our data show that all VHH inhibited binding of b12 to gp140_CN54_ and VHH 1C2, 1B5, 1H9 and A12 prevented the binding of b12 to gp140_CN54_ at concentrations lower than 30 nM.

**Table 2:** Summary of VHH competition with mAb b12 for binding to HIV-1 envelope proteins.

	HIV-1 envelope protein		
VHH	gp140_UG37_	gp140_CN54_	gp120_IIIB_
1C2	+++	++	-
1F10	++	++	-
1E1	++	++	+
1B5	++	++	++
1H9	+++	++	++
1E2	++	++	-
2E7	++	++	+
A12	+++	++	++

The amount of VHH required to reduce the b12 signal by 50% of its maximum was estimated from the respective competition curves. +++ <0.44 µg/mL; ++ 0.44–12 µg/mL; + >12 µg/mL; -,no competition was observed even at the highest amount of VHH.

### Cross-competition assay

**Figure 3:**
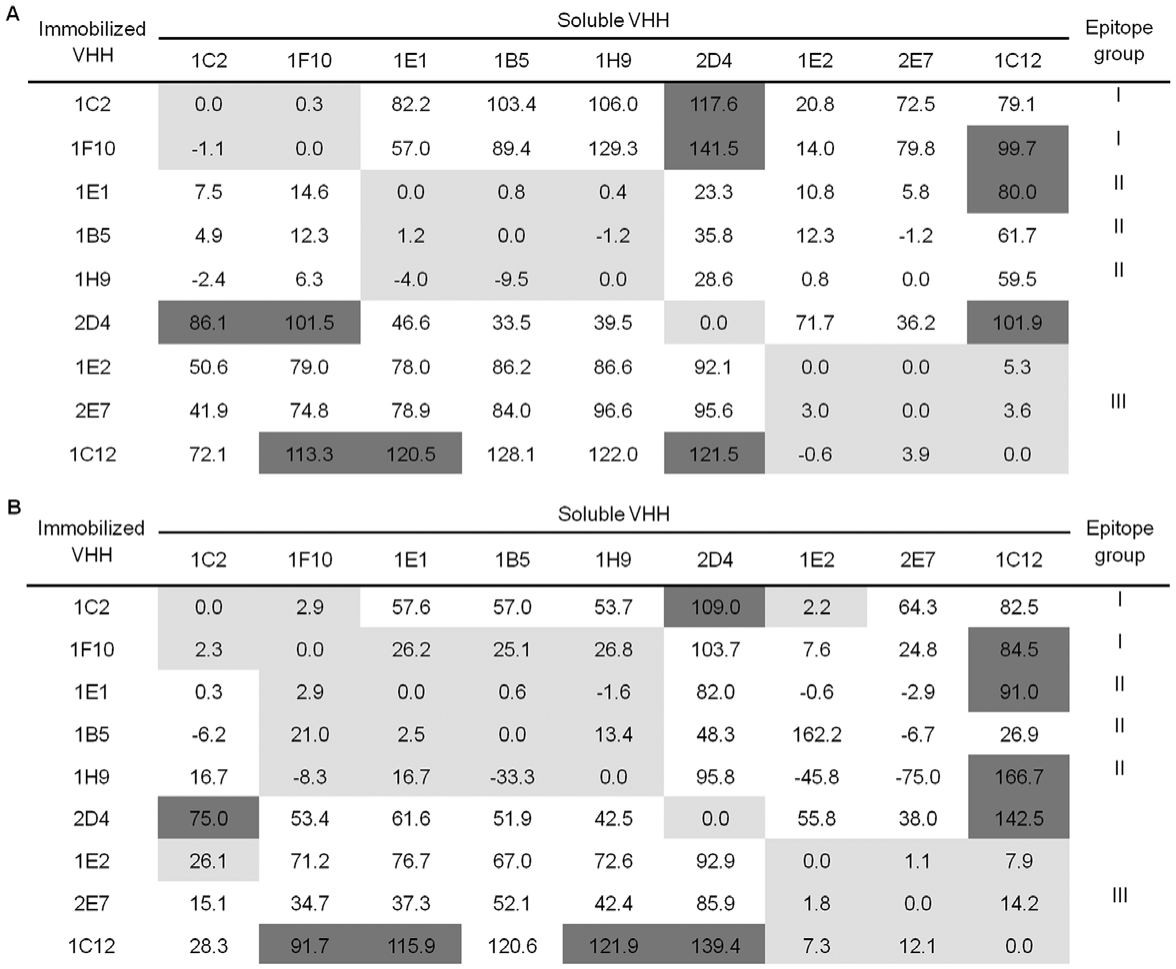
Competition between different VHH for binding toward recombinant envelope proteins. Competition matrix of VHH to gp140_UG37_ (A) and gp140_CN54_ (B). The values shown represent the mean binding of envelope protein in presence of the competitor VHH. The effect of competition is expressed in percent of positivity and marked mutual cross-competition (<30%) are shown by light grey cells and weak mutual cross-competition (>75%) by dark grey cells.

To further characterise the selected VHH, we tested whether they compete with each other or if they can bind to envelope proteins at the same time. For this purpose we applied the competition assay described by Kuroki [52]. Since CD4bs and b12 epitopes differ in various HIV-1 envelope proteins [2], [57], the experiments were performed with both envelope proteins gp140_CN54_ and gp140_UG37_ (Figure 3). Marked mutual cross-competition together with similarity between CDRs was taken as evidence of overlapping epitopes. To define strength of competition we followed the rules described by Tzartos [58]. Although slightly more marked cross-competition reactions were observed for gp140_CN54_ than for gp140_UG37_, the general pattern of cross-competition was similar for both envelope proteins. VHH 2E7 binding to its epitope was hampered by 1E2 and 1C12, and vice versa. Noteworthy is the fact that all three VHH derive from various V genes and differ in CDR3 sequences.

Based on those data and sequence and maturation data the selected VHH were classified into three neutralising groups (I–III) (Figure 3). Group III is composed of 2E7 VHH, which originate from germline V_m_ gene. The other members of this group have been selected by additional rescreening of library 9 (data not shown).

### Neutralisation assay

Functional characterisation of the VHH was assessed in the TZM-bl cell based neutralisation assay developed by Derdeyn *et al*. [59], Wei *et al*. [60] and Li *et al*. [36]. The lowest VHH concentration required to achieve 50% reduction of infectivity (IC_50_) in comparison to a virus control was next determined against a panel of 26 HIV-1 strains from clade A, B, C, A/G and B/C origin. The results presented in the Table 3 show that VHH from different groups have different neutralization profiles. In contrast to the previously described VHH A12, D7 and C8 [35], as well as mAb b12, which showed the most potent activity against HIV-1 subtype B, some of the newly selected VHH were active against subtype C and B/C HIV-1. Overall, VHH 1B5 and 2E7 were the most broadly neutralising VHH demonstrating inhibitory activity against respectively 18 and 21 out of 26 viruses tested, predominantly tier 2 neutralization sensitivity class. VHH 1C2 and 1F10 were active against all subtype A, C and B/C viruses tested, but were inactive against most of subtype B and A/G strains.

**Table 3:** Neutralisation of the different viruses by the VHH and mAb b12.

			IC_50_ in TZM-b1 cells (µg/mL)										
Virus	Subtype	Tier	1C2	1F10	1E1	1B5	1H9	2D4	1E2	2E7	A12	C8	mAb b12
92UG37.A9	A	nd	16	17	•	•	•	•	•	16	•	•	•
MS208.A1	A	1	0.3	0.1	4.1	0.1	0.2	37	4.5	2.9	•	•	0.6
T257-71	CRF02_AG	2	•	•	•	•	•	•	•	45	•	•	•
T266-60	CRF02_AG	2	•	•	•	5.5	8.7	•	15	9.0	•	•	•
T278-50	CRF02_AG	2	•	•	•	10	11	•	•	•	•	•	25
T33-7	CRF02_AG	2	•	38	•	•	•	•	•	38	•	•	•
MN	B	1	6.7	17	•	•	•	•	39	17	0.2	4.5	0.1
IIIB	B	1	•	•	39	6.4	5.6	•	•	10	0.02	0.3	0.04
JRFL	B	nd	•	•	•	48	42	•	•	36	•	•	8.6
6535.3	B	2	•	•	•	30	21	•	•	41	0.1	28	2.5
THRO4156.18	B	2	•	•	•	21	24	•	•	•	6.2	18	0.5
TRJO4551.58	B	2	•	•	•	40	33	•	•	•	16	•	•
96ZM651.02	C	2	27	2.2	34	11	9	•	•	18	0.1	4.3	•
Du156.12	C	2	33	11	•	4.6	37	•	•	8.9	•	•	<1.9
Du422.1	C	2	37	24	•	•	•	•	•	22	•	•	<1.9
ZM197M.PB7	C	2	8.2	5.2	39	16	8.8	•	•	38	6.0	24	7.4
ZM214M.PL15	C	2	6.0	4.3	•	32	•	•	•	22	•	•	<1.9
ZM233M.PB6	C	2	0.2	0.2	•	48	•	•	17	6.4	7.0	38	•
ZM109F.PB4	C	2	1.7	1.7	•	•	•	•	•	•	0.8	38	•
ZM135M.PL10a	C	2	2.6	1.9	•	20	13	•	40	17	•	•	•
CAP45.2.00.G3	C	2	5.8	38	28	7.0	3.7	•	•	30	•	•	<1.9
CH038.12	CRF07_BC	2	2.0	0.8	•	•	•	•	•	13	•	•	<1.9
CH064.20	CRF07_BC	2	4.5	1.5	•	15	22	32	•	20	•	•	•
CH091.9	CRF07_BC	2	14	4.5	•	8.2	38	50	•	36	•	•	•
CH110.2	CRF07_BC	2	6.3	2.6	•	•	40	•	18	16	•	•	•
CH181.12	CRF07_BC	2	2.5	0.8	17	5.9	7	50	•	•	•	•	<1.9
VHH epitope group	I	I	II	II	II			III					

Comparison of neutralization potencies of VHH and mAb b12 in TZM-b1 neutralisation assay against 26 viruses. IC_50_ values above 50 µg/mL are marked as • and IC_50_ values of A12, C8 and mAb b12 are cited from Forsman *et al*. [35].

### Effect of CDR1 and CDR3 mutations on 2E7 activity

As shown above, 2E7 VHH revealed the broadest cross-subtype neutralisation activities, yet its neutralisation potency was lower than b12. Therefore we were interested in determining the influence of single amino acids substitutions in 2E7 VHH on envelope protein binding and neutralisation potency as a start for *in vitro* maturation. We were particularly interested in CDR1 and CDR3 regions since they have been shown to be mainly involved in interaction with antigen [61], [62]. Surprisingly back-mutation to germline residue on position 29 (V29F) seems to enhance binding of the VHH to gp140_CN54_. The alanine scan showed lack of effect of D32A on binding to envelope proteins and better binding of V29A mutant to gp140_CN54_.

Alanine scan of the CDR3 region (Table S1) revealed that the binding to both envelope proteins was unchanged for most VHH mutants. The exceptions were Y98A, Y99A and Y100cA mutants, which bound worse to gp140_UG37_ and gp140_CN54_. Remarkably, mutation of R100bA completely reduced binding of mutant to gp140_CN54_, but not gp140_UG37_.

To verify the influence of the mutations on biological function of the VHH, the neutralisation assays were performed against four viruses (Table 4). The ZM233M.PB6 virus was the most resistant and any mutation tested had an adverse effect on 2E7 potency. In contrast Du156.12 virus was the most sensitive to all mutants except Y98A mutant, which revealed decreased potency against all viruses. Interestingly alanine substitution at position 29 slightly enhanced the potency of 2E7 VHH against 96ZM651.02, 92UG37.A9 and Du156.12, making this residue an interesting start point for future *in vitro* maturation studies. This is in agreement with previously observed enhancement of binding affinities.

**Table 4:** Neutralisation potencies of wild type VHH 2E7 and mutated variants in TZM-b1 neutralisation assay.

			IC_50_ in TZM-b1 cells (µg/ml)					
Virus	Subtype	Tier	Wild type	V29A	Y98A	R100bA	Y100cA	Y102A
92UG37.A9	A	Nd	16	9	28	16	13	12
96ZM651.02	C	2	18	9	23	20	28	14
Du156.12	C	2	9	4	11	5	6	3
ZM233M.PB6	C	2	6	14	45	33	28	23

Comparison of neutralization potencies of VHH 2E7 wild type and 5 mutants against a panel of 4 viruses. IC_50_ values are given in µg/mL. Not done is marked as Nd.

## Discussion

HIV-1 subtype C viruses have become predominant epidemic strains in the world (http://www.unaids.org). The well described broadly neutralizing antibodies of human origins b12, 2G12, 4E10 and 2F5 overall show limited neutralization of subtype C viruses [12], [13], [63], [64]. More recent, potent antibodies PG9, PG16 and VRC01-03, have been selected from blood cells of HIV patients [16], [17], [19], [27]. Nonetheless selection of novel anti HIV-1 neutralizing antibodies is crucial for a better understanding of immune responses to non-subtype B viruses. Further, it is important for the development of better and cheaper microbicides, effective against the most prevalent subtypes, as the recently tested tenofovir gel only reached 39% protection [65].

In the current study, two llamas were immunized with a cocktail of recombinant envelope proteins of HIV-1 subtype A (gp140_UG37_) and B/C (gp140_CN54_) to select VHH against non-subtype B HIV-1 using competitive elution. From the 280 clones tested, 30 clones competed with b12. Sequence analysis revealed 17 different VHH that were clustered into 7 families based on V-, D- and J- genes used during their maturation. Our previous data, described by Forsman *et al*. in 2008 [35] demonstrate that competition between sCD4 and phages results in the release of a phage population enriched in sCD4 competitors. Free gp140 is thought to sample many conformations [66] and only CD4-bound conformation promotes the virus entry process [67]. Thus it is reasonable to assume that during selection, VHH could recognize different envelope protein conformations, and subsequent elution with sCD4, could release not only CD4bs binders, but also VHH that interact with regions involved in transition to CD4-bound conformation and therefore could reveal neutralisation activities.

The lack of crystal structures of envelope proteins in complex with VHH prevents us to exactly localize VHH epitopes. However, four sets of experimental data, i.e. binding to envelope proteins (Table 1), sequences (Fig. 2), cross-competition data (Figure 3) and neutralization data (Table 3), allowed us to categorize the selected VHH into epitope groups. Moreover, current knowledge of envelope protein with sCD4, b12 and F105 structures [68] and VHH interactions with mutated envelope protein helped us to deduce the localisation of VHH epitopes. The structural analysis studies have revealed that both sCD4 and b12 recognize non linear epitopes on the envelope protein, ranging from amino acid 124 to 477, and that F105 binds to a different site than b12 [2], [69]. This information has been applied by the group of Peter Kwong to construct mutant envelope proteins, in which the bridging sheet was tethered to the inner domain [21]. We used both mutated (gp120_Ds2_) and wild type gp120_YU2_ envelope proteins for VHH characterisation. Similarly to most of the antibodies that target the site of CD4 binding [2] the previously selected high affinity VHH A12 and D7 [35] most likely recognize the cavity under the bridging sheets as they bound to gp120_YU2_, but not to gp120_Ds2_.

More recent studies show that VHH A12 and the related VHH D7 [35], [70] most likely bind to an epitope equal or closely related to the epitope recognized by F105 (Chen, in preparation). Since F105 is not a broad neutralizing antibody, this epitope will most likely not yield broad VHH either and thus there is a need for VHH that recognize other epitopes. From the VHH selected in this study, the VHH of group I (represented by 1F10 and 1C2) did not bind to subtype B envelope proteins, showed different competition and neutralization patterns than VHH of groups II and III and therefore they may bind to B20/B21, which forms a loop between the cavity and the outer domain. The VHH belonging to group II (1B5, 1H9, 1E1) most likely recognize the outer domain contact site for CD4 of the envelope protein [2], since these VHH interacted with both gp120_Ds2_ and gp120_YU2_. However, in contrast to b12, VHH 1B5 recognized a larger number of viruses including non B viruses and neutralized in total 18 out of 26 viruses.

Based on these considerations and the 3D structures of envelope proteins we propose that the three major groups of anti-HIV VHH bind to the envelope protein in the cavity below the bridging sheets (A12/D7), the bridging sheet themselves (1F10) and the outer domain (1B5). VHH 2E7, representing group III, bound slightly to gp120_IIIB_, but not to gp120_YU2_ nor to gp120_Ds2_ and had the broadest neutralisation spectrum of HIV-1 subtypes (21 out of 26) of any of the other CD4bs targeting antibodies derived from immunizations. The epitope recognized by VHH 2E7 differs from the antigenic determinants of the 1B5 group as is shown by competition experiments and the various spectra of viruses neutralized by group II and III. Cross-competition between 2E7 and 1E2 or 1C12 VHH probably resulted from alteration of the antigenic determinant conformation by the competing VHH or steric occlusion. To further map the exact location where the VHH bind to the envelope protein, competition assays can be performed with more mAbs of known specificity. Also their binding to two additional Yu-2 mutants, D368R which is not bound by sCD4 or CD4bs mAbs, or I420R which is no longer bound by CD4i specific mAbs, can be tested [18]. The large breadth of VHH 2E7 and 1B5 makes them interesting candidates for further studies. For that reason we carried out a restricted number of mutations on basis of the maturation analysis and this provided us with both an improved neutralising 2E7 VHH variant and insight, which amino acids of CDR1 and CDR3 take part in envelope protein binding.

Although the VHH epitopes still remain to be precisely localized with biophysical methods, our data strongly suggest that the neutralising VHH recognized various epitopes. *In vitro* maturation of 2E7 and 1B5 and construction of biparatopic VHH [71], in particular biparatopic VHH of group I and II or II and III, might broaden virus cross-neutralization and enhance the VHH potency. The effect of bivalency was recently described with a molecule that combined the binding domains of sCD4 and 17b with a 35 amino acid linker. This construct neutralized all 47 strains tested coming from clade A, B, C, D, F, A/E and A/G, 90% with an IC_50_ below 4 µg/ml [72].

The evidence that cell-to-cell transfer may be a major factor in spreading the HIV-1 [28], [73] and the fact that VHH are small enough to operate in the viral synapsis may increase the potential of anti-HIV-1 VHH as functional ingredient in microbicides just as their generally high stability [74]. As microbicides are generally tested on simian-human immunodeficiency virus (SHIV) a small scale assay was performed to evaluate that. 1B5 and 2E7 were active against type B SHIV, 1B5 and 1F10 against type C SHIV (to be published). Finally, the new VHH recognizing three different areas of the CD4bs may provide useful insight into vaccine development.

## Supporting Information

Figure S1 **Binding of CD4 and B12 against recombinant gp140.** Binding of 3 µg/mL sCD4 (solid line) and 100 ng/mL b12 antibody (dashed line) to gp140_UG37_ (black triangle) or gp140_CN54_ (black square) directly coated.
(TIF)

Figure S2 **Binding of VHH to various recombinant envelope proteins.** Binding of VHH to gp120_Ds2_ (black square), gp120 _IIIB_ (dark grey square), gp140_CN54_ (light grey square), gp140_UG37_ (white square) and gp120_YU2_ (dashed). Binding was tested in an ELISA setup and expressed by means of absorbance (A_490_) with subtracted background.
(TIF)

Figure S3 **Inhibition of the binding of the recombinant envelope proteins toward b12 by the VHH.** Binding of HIV-1 envelope proteins gp120 _IIIB_ (dark grey square). gp140_CN54_ (light grey square). gp140_UG37_ (white square) in complex with VHH to immobilized mAb b12. Data are expressed as percent of positivity calculated by equation 100*((A_490sample_−A_490 min_)/(A_490max_−A_490 min_)) where A_490 min_ is a A_490_ of sample without VHH and without gp for particular gp and day of experiment; and A_490max_ is A_490_ of samples without VHH for particular envelope protein and day of experiment.
(TIF)

Table S1 **Binding of 2E7 wild type and mutants to recombinant envelope proteins.** Binding affinities of VHH 2E7 and a panel of its modified variants to HIV-1 envelope proteins gp140UG37 and gp140CN54 as measured by ELISA. ^a^ Binding affinities were calculated as the antibodies concentration at half-maximal binding. ^b^ Key: n.b., no binding was determined.
(XLS)
