# Supplementary figures and images for: Llama Antibody Fragments Recognizing Various Epitopes of the CD4bs Neutralize a Broad Range of HIV-1 Subtypes A, B and C
Source: PLoS One. 2012 Mar 15;7(3):e33298. doi: 10.1371/journal.pone.0033298 (PMC3305327; doi:10.1371/journal.pone.0033298)

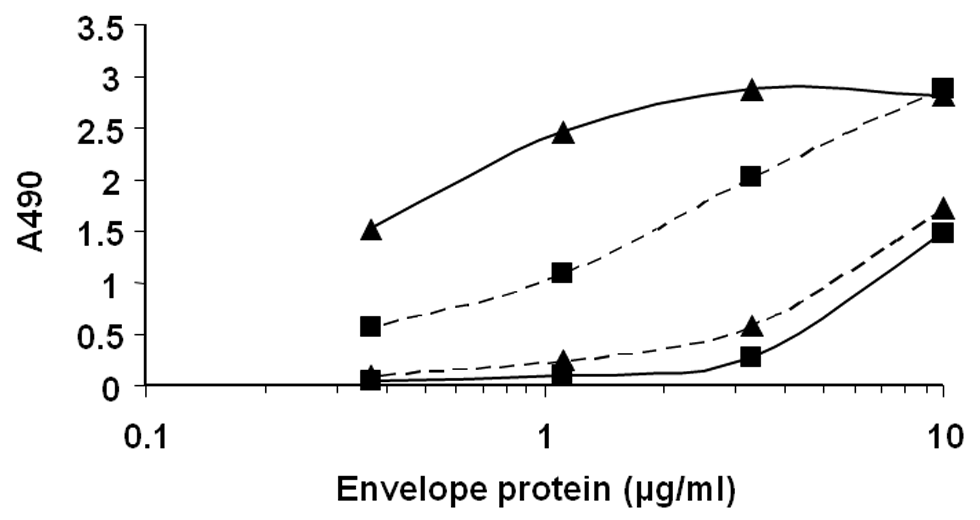

Supplement: Figure S1 — Binding of CD4 and B12 against recombinant gp140. Binding of 3 µg/mL sCD4 (solid line) and 100 ng/mL b12 antibody (dashed line) to gp140UG37 (black triangle) or gp140CN54 (black square) directly coated. (TIF) [file pone.0033298.s001.tif]

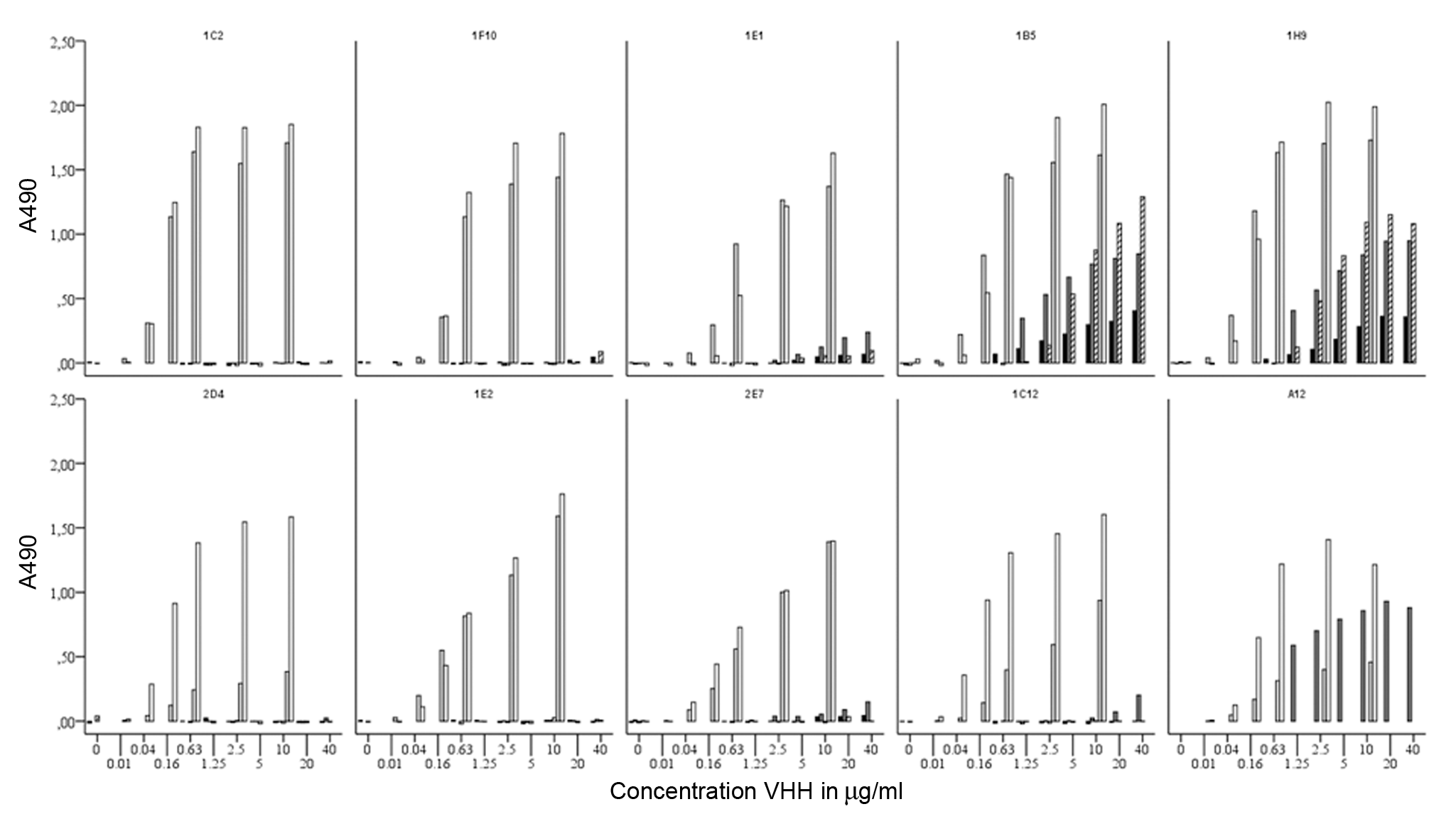

Supplement: Figure S2 — Binding of VHH to various recombinant envelope proteins. Binding of VHH to gp120Ds2 (black square), gp120 IIIB (dark grey square), gp140CN54 (light grey square), gp140UG37 (white square) and gp120YU2 (dashed). Binding was tested in an ELISA setup and expressed by means of absorbance (A490) with subtracted background. (TIF) [file pone.0033298.s002.tif]

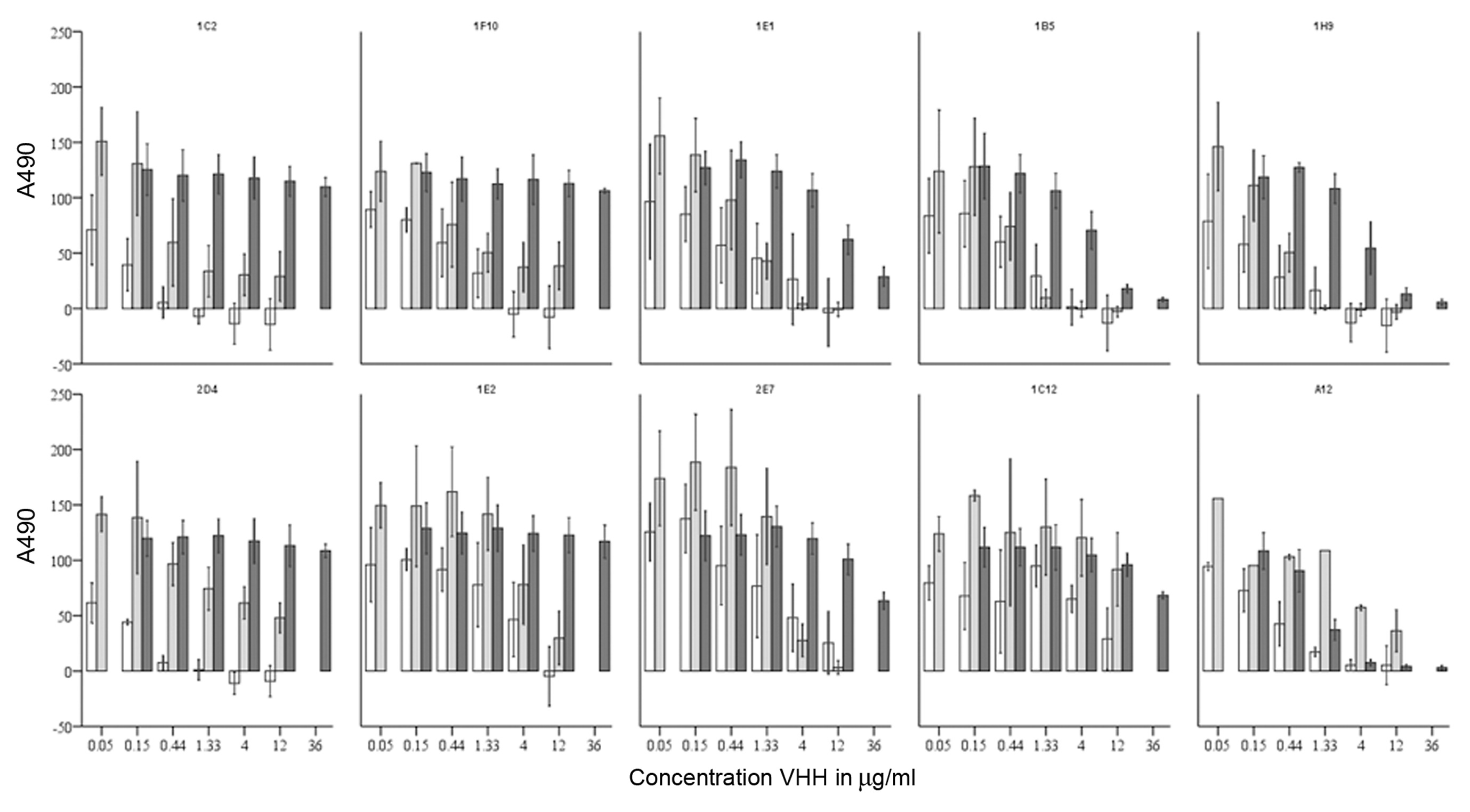

Supplement: Figure S3 — Inhibition of the binding of the recombinant envelope proteins toward b12 by the VHH. Binding of HIV-1 envelope proteins gp120 IIIB (dark grey square). gp140CN54 (light grey square). gp140UG37 (white square) in complex with VHH to immobilized mAb b12. Data are expressed as percent of positivity calculated by equation 100*((A490sample−A490 min)/(A490max−A490 min)) where A490 min is a A490 of sample without VHH and without gp for particular gp and day of experiment; and A490max is A490 of samples without VHH for particular envelope protein and day of experiment. (TIF) [file pone.0033298.s003.tif]
